# Predicting 3D lip shapes using facial surface EMG

**DOI:** 10.1371/journal.pone.0175025

**Published:** 2017-04-13

**Authors:** Merijn Eskes, Maarten J. A. van Alphen, Alfons J. M. Balm, Ludi E. Smeele, Dieta Brandsma, Ferdinand van der Heijden

**Affiliations:** 1 Dept of Head and Neck Oncology and Surgery, Netherlands Cancer Institute, Amsterdam, the Netherlands; 2 MIRA Institute of Biomedical Engineering and Technical Medicine, University of Twente, Enschede, the Netherlands; 3 Dept of Oral and Maxillofacial Surgery, Academic Medical Center, Amsterdam, the Netherlands; 4 Dept of Neuro-Oncology, Netherlands Cancer Institute, Amsterdam, the Netherlands; 5 Dept of Neurology, Slotervaart Hospital, Amsterdam, the Netherlands; University of Houston, UNITED STATES

## Abstract

**Aim:**

The aim of this study is to prove that facial surface electromyography (sEMG) conveys sufficient information to predict 3D lip shapes. High sEMG predictive accuracy implies we could train a neural control model for activation of biomechanical models by simultaneously recording sEMG signals and their associated motions.

**Materials and methods:**

With a stereo camera set-up, we recorded 3D lip shapes and simultaneously performed sEMG measurements of the facial muscles, applying principal component analysis (PCA) and a modified general regression neural network (GRNN) to link the sEMG measurements to 3D lip shapes. To test reproducibility, we conducted our experiment on five volunteers, evaluating several sEMG features and window lengths in unipolar and bipolar configurations in search of the optimal settings for facial sEMG.

**Conclusions:**

The errors of the two methods were comparable. We managed to predict 3D lip shapes with a mean accuracy of 2.76 mm when using the PCA method and 2.78 mm when using modified GRNN. Whereas performance improved with shorter window lengths, feature type and configuration had little influence.

## Introduction

Treatment choice for oral cavity carcinoma still depends on the subjective judgements of treating physicians and multidisciplinary tumour boards. The primary choice of treatment will normally be surgery, with or without adjuvant radiotherapy [1]. If the functional consequences of surgery would reduce the quality of life to an unacceptable extent, the tumour is considered ‘functionally inoperable’ [2], and organ-sparing chemoradiation treatment will provide a better alternative. Yet, ‘functional inoperability’ is a subjective label and as such not very reliable [3].

To predict functional consequences more objectively, we have been developing a virtual-therapy tool that comprises a patient-specific biomechanical model of the oral cavity and oropharynx [4]. To further individualise this model, we proposed implementing electromyographic (EMG) signals to estimate volunteer-specific muscle activations during specific tongue movements. Since surface EMG (sEMG) of the tongue is difficult to perform, we decided first to look at lip shapes, which are easier to capture in 3D images, while their underlying muscle activation patterns are easy to assess with sEMG, yet the facial musculature is still complex enough to prove our concept.

Most research efforts with facial EMG have focussed on speech interfaces, mostly silent-speech interfaces [5] and multimodal speech synthesis models [6]. Their general aim has been to categorise phonemes, words, articulatory features, or gestures by facial and tongue EMG signals [7–14]. Honda et al. [15] and Lucero & Munhall [16] have both published on predicting lip shapes. Honda et al. [15] used video imaging to estimate lip shapes, but the images were in 2D, and their model did not account for jaw movements. Lucero & Munhall’s [16] finite-element model (FEM) of the face and lips estimated 3D lip positions, but their lip marker correlation coefficients were relatively low (mean<0.71).

We are now taking a step forward by investigating two methods to estimate 3D lip shapes. Our first objective was to show that facial sEMG can adequately estimate volunteer-specific 3D lip shapes. If sEMG conveys sufficient information to estimate lip shapes, we could use that information together with simultaneous video recordings of the pertaining motions to train a neural control model for the activation of a personalised biomechanical model that in the end will present the effects of treatment in a virtual-therapy environment. Furthermore, this could perhaps bring us closer to solving the load-sharing problem of inverse dynamics [17]. Finally, accurate sEMG-based lip modelling would also be helpful in other fields, such as silent-speech interfaces and multimodal speech synthesis [5,6,11].

Our second objective was to see if we could identify any volunteer-independent settings for sEMG feature extraction, which would greatly benefit our future application: an individualised biomechanical simulation model for lip and oral cancer patients. Not having to optimise the settings per patient would save us a lot of time and effort.

## Materials and methods

### Volunteers and data acquisition

To test reproducibility, we recruited five volunteers (four males and one female) aged between 24 and 25. In our recruiting e-mail, we briefly explained about our experiment and on the test day itself, we once again informed them of the procedure and of their rights as volunteers, including the right to withdraw at any moment without stating a reason. All volunteers gave their informed consent and their approval for publication of anonymised results. This experiment was approved by the Medical Research Ethics Committee of the Netherlands Cancer Institute and conducted in accordance with Dutch legislation, including the Agreement on Medical Treatment Act, Personal Data Protection Act, and the Code of Conduct for Responsible Use of the Federa (Dutch Federation of Biomedical Scientific Societies).

**Fig 1:**
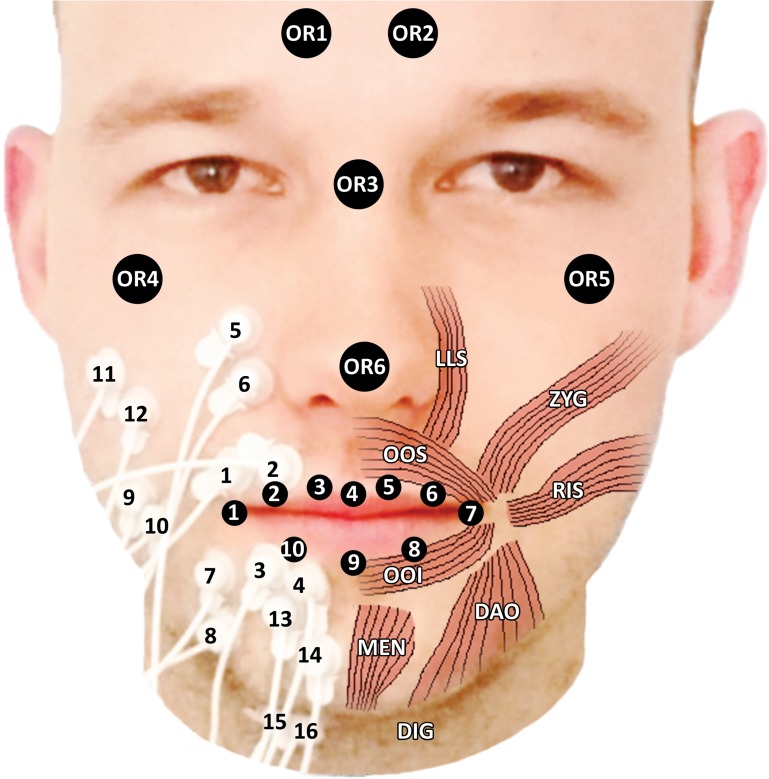
Surface electrode and facial marker positions. Volunteer with sEMG electrodes in bipolar configuration placed on the zygomaticus major, risorius, orbicularis oris superior and inferior, mentalis, depressor anguli oris, levator labii superioris, and digastric muscles, and showing ten markers on the lips and six infraorbitally, supraorbitally, and on the nose (muscle anatomy adapted from [19]).

With a black skin marker, we marked ten points on the lips for measuring 3D lip positions and six on the face (two infraorbitally, two supraorbitally, and two on the nose) to compensate for head movements, see Fig 1. Our camera set-up consisted of two consumer cameras (Casio® EX-FC100), which we calibrated with a 10x10x10 cm wireframe cube with 27 nodes at known positions before placing it in front of the volunteer. To quantify the measurement error of our camera measurement device, we calculated the root mean square (RMS) of the distances between the actual 3D node positions and their 3D positions as calculated from the two stereo images. Using a leave-one-out method, we calibrated with 26 nodes and rotated the remaining node so that we obtained 27 error distances, from which we calculated the RMS measurement error, being 0.63 mm.

Led by human lip anatomy and a paper by Lapatki et al. [18], we decided to perform sEMG measurements of the zygomaticus major (ZYG), the risorius (RIS), the orbicularis oris superior (OOS) and inferior (OOI), the mentalis (MEN), the depressor anguli oris (DAO), and the levator labii superioris (LLS) muscles. We further included the digastric muscle (DIG) to represent jaw opening in our model.

For performing sEMG measurements, we used a Porti-system from TMSi (Oldenzaal, the Netherlands) with sintered disc-shaped sEMG micro-electrodes (1.5 mm diameter, Ag/AgCl) with shielded cables, see Fig 1. As the size of the electrodes prohibited interelectrode distances (IEDs) smaller than 10 mm, we used 10-mm IEDs. Because of individual differences in face dimensions, we could not use a ruler to apply the electrodes, so we placed them according to Fig 1 and then fine-tuned their positions by searching for maximum sEMG output. Finally, we placed a self-adhesive common ground reference electrode on the left wrist.

### Instructions to volunteers

**Fig 2:**
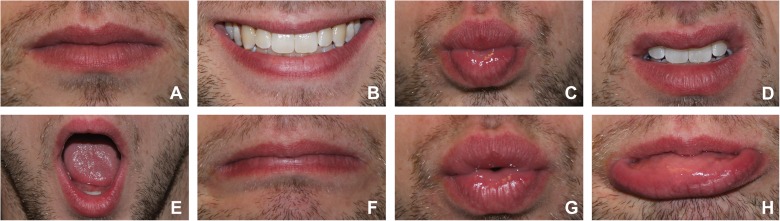
Rest pose and seven facial expressions as instructed to the volunteers. A: rest, B: voluntary smiling, C: pursed lips, D: raised upper lip, E: mouth open, F: depressed mouth corners, G: blowing, H: pouting.

We asked our volunteers to adopt thirteen poses (including a rest pose) by making seven facial expressions (voluntary smiling, pursed lips, raised upper lip, mouth open, depressed mouth corners, blowing, and pouting; see Fig 2) and five vowel sounds (/a/, /e/, /i/, /o/, /u/) for four seconds each, in random order. Between each pose, they were to adopt a rest pose with closed mouth and relaxed muscles to serve as our reference when defining the magnitude of marker position displacements in the other poses, since only displacements can be inferred from sEMG signals.

The seven facial expressions correspond with isolated facial muscle contractions [18]. For non-trained volunteers, it is very difficult to perform the poses without any cocontractions of other muscles. Since multiple variables are involved in model training, these cocontractions are embedded in the model and automatically disentangled during the prediction phase. We took pictures of each pose with both cameras and simultaneously performed sEMG measurements, repeating our data acquisition four times with a pause between repetitions.

### Data processing and analysis

After manually selecting the marker positions in the images and coming up with a set of 40 lip pixel coordinates for each pose (ten markers, 2D positions with two cameras), we reconstructed these coordinates into a vector, **X**(*p*,*r*) ∈ ℝ^30^, which held the 30 coordinates of the ten markers in 3D space. Here, *p* = 0, ···, 12 gives the pose number, and *r* = 1, ···, 5 the repetition number. Referring to the facial markers, we used them to register all poses to the same reference frame to correct for head motion.

Our manual selection of the image points induced an error. To assess this error, as well as the error induced by imperfect head motion compensation, we estimated the RMS of the differences between two selection runs, the so-called intraobserver error (*e*_*obs*_). Let *x*_*m*,*1*_ (*p*), *m* = 1, ···, 30 denote the coordinates of the marker position of pose *p* in the first selection run and *x*_*m*,*2*_ (*p*) the ones in the second run, then we defined *e*_*obs*_ as the RMS of the Euclidean length of the differences, calculating it over the ten markers and thirteen poses (including the rest pose) as follows:

$$e_{obs}=\sqrt{\frac{1}{130}\sum_{p=0}^{12}\sum_{m=1}^{30}{\left(x_{m,1}^{}\left(p\right)-x_{m,2}^{}\left(p\right)\right)}^{2}}$$ (1)

The RMS intraobserver error turned out to be *e*_*obs*_ = 2.34 mm, which implies that the measurement error (0.63 mm) will not have had a large impact on our position estimation. After all, the measurement and observer errors are independent. Therefore, the total of errors follows from the root of the sum of squares. Neglecting the measurement error would result in an underestimation of the total error of √(2.34^2^ / (0.63^2^ + 2.34^2^)) = 0.97.Notably, the intraobserver error involved two selection runs, which implies that the error of a single run would equal *e*_*obs*_ /√2, with the assumption of uncorrelated errors.

To assess the magnitude of marker position variations, we calculated the RMS of the Euclidean distance between the ten markers of a pose and the corresponding markers in the rest position (*p* = 0), which we then averaged over the twelve poses and the five repetitions:

$$d_{RMS}=\sqrt{\frac{1}{600}\sum_{r=1}^{5}\sum_{p=1}^{12}\sum_{m=1}^{30}{\left(x_{m}\left(p,r\right)-x_{m}\left(0,r\right)\right)}^{2}}$$ (2)

Here, *x*_*m*_ (*p*,*r*) denotes the *m*^th^ element from the vector **X**(*p*,*r*). We also corrected the magnitude of variation (*d*_*RMS*_) for the intraobserver error (*e*_*obs*_) between two selection runs to correct the two selections *x*_*m*_ (*p*,*r*) and *x*_*m*_ (0,*r*) as follows:

$$d_{c}=\sqrt{d_{RMS}{}^{2}-e_{obs}{}^{2}}$$ (3)

To estimate lip positions from our sEMG recordings, we used two methods: nonlinear regression with principal component analysis (PCA) and nonlinear regression with a modified version of general regression neural network (GRNN). We have described these methods below. To assess the accuracy of the positions $\hat{X}(p,r)$ as estimated by both regression methods, we calculated the RMS of the Euclidean length of the differences between marker positions of the estimated pose and the reference pose:

$$e_{RMS}=\sqrt{\frac{1}{600}\sum_{r=1}^{5}\sum_{p=1}^{12}\sum_{m=1}^{30}{\left({\hat{x}}_{m}\left(p,r\right)-x_{m}\left(p,r\right)\right)}^{2}}$$ (4)

where ${\hat{x}}_{m}\;(p,r)$ is the *m*^th^ element of the vector $\hat{X}(p,r)$. We corrected this *e*_*RMS*_ for the manual selection error by applying the factor *e*_*obs*_ /√2, introducing the factor 1/√2 because the *e*_*obs*_ was based on two manual selections runs, whereas in Eq (4), we have only one selection run:

$$e_{c}=\sqrt{e_{RMS}{}^{2}-\frac{1}{2}e_{obs}{}^{2}}$$ (5)

To compare between volunteers, we defined the ratio of the error to position deviation (*e*_*r*_) as follows:

$$e_{r}=\frac{e_{c}}{d_{c}}$$ (6)

To compare our results with literature [16], we calculated correlation coefficients between ${\hat{x}}_{m}\;(p,r)$ and *x*_*m*_ (*p*,*r*) per volunteer and per coordinate, coming up with a total of 30 correlation coefficients, which we then averaged to find the mean correlation coefficient of each volunteer.

#### sEMG preprocessing

The sEMG signals were recorded with a sample frequency of 2048 Hz. We applied a fourth-order Butterworth bandpass filter with 15 and 500 Hz cut-off frequencies. Thanks to the actively shielded electrode cables, which significantly reduced the mains interference and motion artefacts, we found no significant AC power line interference. Therefore, we omitted a 50 Hz notch filter.

We recorded the signals in two configurations: unipolar and bipolar, extracting four types of sEMG features: mean absolute value (MAV), root mean square (RMS), waveform length (WL), Willison amplitude (WAMP) with thresholds at *s*_*lim*_ = 10 mV and *s*_*lim*_ = 20 mV. Table 1 shows the equations for all features, which we chose because they had performed well in a recent experiment [20].

**Table 1:** sEMG feature types and their equations.

Feature	Equation
MAV	$g(j)=\frac{1}{N}\sum_{i=j-\frac{1}{2}(N-1)}^{j+\frac{1}{2}(N-1)}\left|s(i)\right|$
RMS	$g(j)=\sqrt{\frac{1}{N}\sum_{i=j-\frac{1}{2}(N-1)}^{j+\frac{1}{2}(N-1)}s{\left(i\right)}^{2}}$
WL	$g(j)=\sum_{i=j-\frac{1}{2}(N-1)}^{j+\frac{1}{2}(N-1)-1}\left|s(i+1)-s(i)\right|$
WAMP *s*_lim_ = 10 *s*_lim_ = 20	$\begin{array}{l} g(j)=\sum_{i=j-\frac{1}{2}(N-1)}^{j+\frac{1}{2}(N-1)-1}\left[v\left(\left|s(i+1)-s(i)\right|\right)\right]\\ v(s)=\{\begin{array}{c} 1,\;\text{if}\;s\geq s_{\lim}\\ 0,\;\text{otherwise} \end{array} \end{array}$

We defined the features for six window lengths: 50, 100, 150, 200, 250, and 300 ms and used a sliding window with maximum overlap (all-but-one sample) to calculate the features: if there were *n* sEMG samples in a record and the window length was *p* samples, the resulting EMG feature record had *n – p +* 1 samples. Our reason for using a maximum overlap was to get a maximum number of feature vectors per record. Notwithstanding the inevitable autocorrelation within the features, maximising the number of features results in better performance of both estimation methods.

We decided to truncate the calculated features of approximately four seconds to three seconds exactly (i.e. 6145 samples) to achieve an equal amount for each feature set **g**(*i*,*p*,*r*). There are 60 feature sets **g**(*i*,*p*,*r*) containing the features of 8 sEMG channels with *i* = 1, ···, 6145 (the samples), *p =* 1, ···, 12 (the poses), and *r* = 1, ···, 5 (the repetitions).

#### Principal component analysis based estimation

We trained our PCA-based regression method using a database that included observed marker positions and associated feature vectors. As we used static poses, we considered the raw sEMG signals to be stationary during each pose. Consequently, the statistics of features calculated for a sliding window were considered constant. Therefore, for each lip position **X**(*p*,*r*), we needed only one sEMG feature vector rather than the whole set of 6145 sEMG feature vectors **g**(*i*,*p*,*r*), thus avoiding a huge dimension of the measurement space.

We first averaged the vectors in a set over the time samples to yield a single 8D feature vector **ḡ**(*p*,*r*). As we figured we could not perform a linear mapping from this 8D feature space to the 30D position space, we decided to apply nonlinear regression. We did try using linear regression at first, but ended up with large errors. The simplest way of approximating a nonlinear mapping is using a truncated Taylor series expansion of only the quadratic terms. So, to implement nonlinear regression, we augmented these feature vectors with the ½×8×9 = 36 quadratic terms that could be formed from the eight elements in **ḡ**(*p*,*r*), thus obtaining a set of 44D data vectors **ḡ**_*aug*_(*p*,*r*). Next, we concatenated this vector with the 30 coordinates **X**(*p*,*r*) of the pose, which gave us the following 74D vector **z**(*p*,*r*):

$$z\left(p,r\right)=\left[\begin{array}{c} X\left(p,r\right)\\ {\bar{g}}_{aug}(p,r) \end{array}\right]\;\mathrm{with}\;\begin{array}{l} p=1,\cdots ,12\;(\text{poses})\\ r=1,\cdots ,5\;(\text{repetitions}) \end{array}$$ (7)

The training of the PCA model was combined with cross-validation to avoid any performance evaluation bias. The PCA model was trained with pooled data from four repetitions of twelve poses each. Testing was done on the remaining repetition. We reiterated this procedure four times while rotating the five repetition sets in the training pool and the test set. Each training pool comprised a 74×48 matrix **Z**_*train*_, the columns of which were vectors **z**(*p*,*r*), with a corresponding test set comprising a 74×12 matrix **Z**_*test*_.

Before developing the PCA model, we first normalised our data with respect to the mean and variance because there were two different physical dimensions and units. For each of the 74 elements in the training set, the average and sample variance were calculated. These two parameters were used to shift and scale the data such that the average was zero and the variance was one. We also performed this operation on the test set.

The PCA model **Y** is a 74×*D* matrix, containing the *D* most dominant eigenvectors **y**_*d*_ that result from **Z**_*train*_ **Z**^*T*^_*train*_ **y**_*d*_ = λ_*d*_ **y**_*d*_, where λ_*d*_ are the eigenvalues. We normalised the eigenvectors, i.e. the principal components, to get **YY**^*T*^ = **I** and sorted the eigenvectors **y**_*d*_ in **Y** = [ **y**_1_, ···, **y**_*d*_ ] to get the condition λ_*d*_ ≥ λ_*d*+1_ for the corresponding eigenvalues.

PCA is basically an encoding/decoding method. Any data vector from the test set **Z**_*test*_, say **z**_*test*_, could be encoded into a lower *D*-dimensional coefficient vector **b**:

$$b=Y^{T}z_{test}$$ (8)

Decoding from **b** uses the same model:

$$z_{test}\approx Yb$$ (9)

Eqs (8) and (9) were not directly helpful in estimating lip positions. In the test set, we wanted to estimate lip positions from the sEMG features, so we could use only that part of the vector **z**_*test*_ that contained the sEMG features. We adapted Eq (9) accordingly and defined the submatrix **Y**_*g*_ of **Y**, which contained the sEMG features only (the lower 44 rows of **Y**). We then had:

$${\bar{g}}_{aug}=Y_{g}b+v$$ (10)

Where **v** contained the residuals that represented the approximation error in Eq (9), and **ḡ**_*aug*_ was the part of **z**_*test*_ that contained the 44 sEMG features. We regarded Eq (10) as a linear observation model of **b**, **Y**_*g*_ being the observation matrix and **v** the observation noise. Least Square Error (LSE) estimation of **b** is then straightforward [21]:

$${\hat{b}}_{LSE}={\left(Y_{g}^{T}Y_{g}\right)}^{-1}Y_{g}^{T}{\bar{g}}_{aug}$$ (11)

${\hat{b}}_{LSE}$ being the estimated coefficient vector. From that, we could estimate the full vector **z**_*test*_, including the 30 lip position coordinates **X** by applying Eq (9):

$${\hat{z}}_{LSE}=Y{\hat{b}}_{LSE}$$ (12)

Undoing the normalisation finalised the estimation.

An extension of this estimation of **b** is the Minimum Mean Square Error (MMSE) estimation. This method exploits the prior knowledge that the PCA coefficients are uncorrelated, with zero means. The covariance matrix **C**_*b*_ of **b** is diagonal with diagonal elements λ_*d*_. With uncorrelated residuals **v**, the covariance matrix is proportional to the unity matrix **C**_*v*_ *= σ*_*v*_^*2*^ **I**. The unbiased MMSE estimate ${\hat{b}}_{MMSE}$, based on the sEMG features, then follows [21]:

$${\hat{b}}_{MMSE}={\left(Y_{g}^{T}Y_{g}+\sigma_{v}^{2}C_{b}^{-1}\right)}^{-1}Y_{g}^{T}{\bar{g}}_{aug}$$ (13)

Obviously, when *σ*_*v*_ is set to zero, ${\hat{b}}_{MMSE}$ equals ${\hat{b}}_{LSE}$. So, the MMSE estimate encompasses the LSE estimate, and there is no need to treat it separately.

#### Extended general regression neural network estimation

The second regression method is an extension of the general regression neural network (GRNN). GRNN is a nonlinear interpolation method based on Parzen kernel density models [22]. We combined the design and evaluation of GRNN with cross-validation in the same way as outlined above. First, we defined a linear index over the poses and repetitions: $c\overset{def}{=}12(r-1)+p$. Assuming the vectors **X**_*c*_ and **ḡ**_*c*_ are associated, we had a population of pairs available in a training pool {**(X**_*c*_,**ḡ**_*c*_*) | c =* 1, ···, 48}. Given a new sEMG vector **ḡ**, GRNN estimates the associated vector $\hat{X}$ by:

$$\hat{X}=\sum_{c=1}^{48}w_{c}X_{c}\;\text{with}:\;{\text{w}}_{c}=\frac{s\left(\bar{g},{\bar{g}}_{c}\right)}{s\left(\bar{g},{\bar{g}}_{1}\right)+s\left(\bar{g},{\bar{g}}_{2}\right)+\ldots +s(\bar{g},{\bar{g}}_{52})}$$ (14)

where *s*(**ḡ**, **ḡ**_*c*_) is a similarity measure between **ḡ** and **ḡ**_*c*_ derived from a Parzen estimate of the underlying probability density. We replaced the Parzen kernel that uses isotropic Gaussians based on Euclidean distances with the likelihood function *p*(**g** | *c*), assuming non-isotropic Gaussians with pose-dependent Mahalanobis distances. This alteration of the standard GRNN would induce better adaptations to the feature vectors’ statistical properties. We defined all 48 poses in a training pool as individual classes. For each class, a feature set **g**(*i*,*p*,*r*)was available, which we used to train the likelihood function *p*(**g** | *c*). In the assumption of normal distributions for the likelihood function *p*(**g** | *c*) = *N* (**g – μ**_*c*_, **C**_*c*_), learning boils down to estimating the mean **μ**_*c*_ and the covariance matrix **C**_*c*_, as in ${\hat{\mu }}_{c}={\bar{g}}_{c}$. We defined the similarity measures associated with a new vector **ḡ** as follows:

$$s(\bar{g},{\bar{g}}_{c})=N(\bar{g}-{\bar{g}}_{c},\alpha^{2}C_{c}+\gamma I)$$ (15)

The introduction of factor *α*^2^ improved the generalisation capability. For each pose, only four repetitions in a training pool were available. Therefore, poses were not well populated in the 8-dimensional feature space. By spreading the Gaussian kernels with the factor *α*^2^, we increased the overlap between kernels. We added the term *γ***I** to improve numerical stability, but the choice of *γ* (around 10^−6^) was not critical.

For each regression method, we determined the best performing combination of feature type, window length, and configuration (of 60 possible combinations) using the cross-validation technique mentioned above. With the PCA method, we evaluated the parameter *σ*_*v*_ for each combination over the range of *σ*_*v*_ = 0, 0.05, ···, 0.3 and the PCA dimension *D* over the range of *D* = 1, 2, ···, 48. With the GRNN method, we analysed the parameter *α* over the range of *α* = 1, 2, ···, 10.

To estimate the mean optimal settings, we averaged the error values *e*_*c*_ over the five volunteers and looked which settings gave the minimum error:

$$\begin{array}{l} e_{c,average}(conf,feat,win,D,\sigma_{v})=\frac{1}{5}\sum_{vol=1}^{5}e_{c}(vol,conf,feat,win,D,\sigma_{v})\\ e_{c}{}_{,\min}=\underset{conf,feat,win,D,\sigma_{v}}{\min}e_{c,average}(conf,feat,win,D,\sigma_{v}) \end{array}$$ (16)

In the GRNN method, we interchanged the parameters *D* and *σ*_*v*_ with *α*.

We applied the one-sided paired Student’s T-test to check for significant differences between the PCA-based regression methods and to test for significant differences between volunteer-independent and volunteer-specific parameters. After all, since the PCA-LSE is in fact included in the PCA-MMSE at *σ*_*v*_ = 0, it can never be better than the PCA-MMSE. The same holds true for volunteer-specific parameters, which will always outperform or be equivalent to volunteer-independent parameters.

We compared the GRNN with the PCA-MMSE using the two-sided paired Student’s T-test, because we did not know whether the GRNN method would perform better or worse than the PCA-based regression method. Finally, we performed a repeated-measures ANOVA test to look for statistically significant influences of the various sEMG feature extraction settings and parameters.

## Results

**Fig 3:**
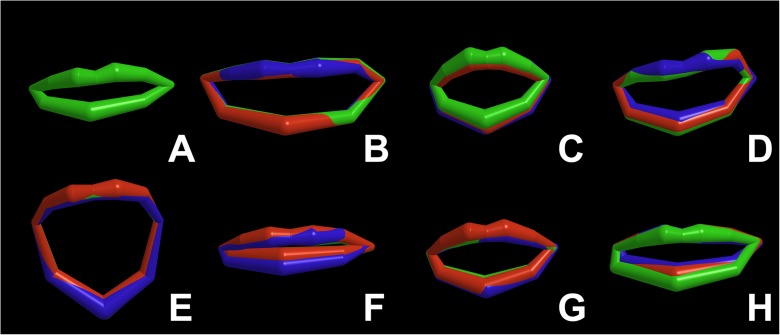
Rest pose and seven facial expressions in 3D of volunteer 5 averaged over the five repetitions. **GREEN: lip shapes tracked by 3D image reconstruction using a stereo camera set-up. RED: lip shapes estimated by the PCA-based nonlinear regression. BLUE: lip shapes estimated by the extended GRNN-based nonlinear regression.** A: rest (not estimated), B: voluntary smiling, C: pursed lips, D: raised upper lip, E: mouth open, F: depressed mouth corners, G: blowing, H: pouting.

Fig 3 gives an idea of the accuracy showing the 3D lip shapes of volunteer 5 with volunteer-specific settings for both PCA-MMSE (in red) and modified GRNN (in blue). Table 2 presents the optimal results of the five individual volunteers for both methods. Table 3 shows the results for the mean optimal settings as calculated by Eq (16). Both with volunteer-specific (*P* << 0.01) and with volunteer-independent settings (*P* << 0.01), the PCA-MMSE method performed significantly better than the PCA-LSE method. However, we found no significant difference between the modified GRNN method and the PCA-MMSE method (volunteer-specific settings: *P* ~ 0.17 and volunteer-independent settings: *P* ~ 0.99), nor did we find any significant difference between the volunteer-specific settings and the volunteer-independent settings (PCA-LSE: *P* ~ 0.82, PCA-MMSE: *P* ~ 0.15, GRNN *P* ~ 0.06). In the PCA-based estimation, dimension *D* (*P* << 0.01), and parameter *σ*_*v*_ (*P* << 0.01) were both statistically significant, whereas feature type (*P* ~ 0.14), window length (*P* ~ 0.06), and configuration (*P* ~ 0.06) were not. In the modified GRNN-based estimation, parameter α (*P* << 0.01) and window length (*P* << 0.01) were statistically significant, whereas feature type (*P* ~ 0.07) and configuration (*P* ~ 0.58) were not. The averaged data showed somewhat lower error measures *e*_*c*_ and *e*_*r*_ when we used the PCA method. Nevertheless, with volunteer-specific settings, the GRNN method performed better in four volunteers.

**Table 2:** The lowest error values for *e*_*c*_ and *e*_*r*_ by volunteer with the corresponding settings.

Volunteer	*e*_*c*_ (mm)		*e*_*r*_		*ρ*		Configuration		Feature ^a^		Window (ms)		*D*	*σ*_*v*_	*α*
	PCA	GRNN	PCA	GRNN	PCA	GRNN	PCA	GRNN	PCA	GRNN	PCA	GRNN	PCA		GRNN
1	2.71	2.69	0.24	0.24	0.90	0.90	Uni	Uni	1	1	200	200	10	0.05	3
2	3.00	3.12	0.29	0.31	0.88	0.86	Bi	Bi	2	3	300	150	17	0.25	5
3	2.41	2.25	0.23	0.22	0.91	0.92	Uni	Uni	1	1	300	50	12	0.10	2
4	1.99	1.42	0.23	0.17	0.91	0.94	Uni	Bi	1	4	300	50	7	0.05	2
5	2.94	2.39	0.33	0.27	0.87	0.90	Uni	Uni	1	5	100	200	6	0.05	3

a. Optimal features are represented by numbers as follows: 1. WAMP *s*_*lim*_ = 10 mV; 2. WAMP *s*_*lim*_ = 20 mV; 3. WL; 4. MAV; 5. RMS.

**Table 3:** The error values for *e*_*c*_ and *e*_*r*_ by volunteer with the mean optimal settings, i.e. volunteer-independent settings (see Eq (16)).

Volunteer	*e*_*rms*_ (mm)		*e*_*c*_ (mm)		*e*_*r*_		*ρ*	
	PCA^a^	GRNN^b^	PCA^a^	GRNN^b^	PCA^a^	GRNN^b^	PCA^a^	GRNN^b^
1	3.18	3.68	2.71	3.28	0.28	0.33	0.90	0.89
2	3.94	3.58	3.57	3.17	0.36	0.32	0.85	0.85
3	2.95	2.95	2.45	2.44	0.25	0.25	0.91	0.91
4	2.60	2.18	2.01	1.42	0.20	0.14	0.91	0.94
5	3.40	3.80	2.97	3.42	0.30	0.35	0.88	0.86
Averaged	3.21	3.24	2.74	2.75	0.28	0.28	0.89	0.89
Corrected Average	2.76	2.78

a. PCA settings: configuration = unipolar; feature = WAMP *s*_*lim*_ = 10 mV; window length = 300 ms; *D* = 9; *σ*_*v*_ = 0.05.

b. GRNN settings: configuration = bipolar; feature = MAV; window length = 50 ms; *α* = 2

We found the lowest mean error in the unipolar configuration when estimating positions with PCA and in the bipolar configuration when using GRNN. However, three volunteers performed better in the unipolar configuration. When we interchanged configurations, the corrected error with mean optimal settings (*e*_*c*,min_) became 3.78 mm when using PCA and 3.01 mm when using GRNN. The PCA method showed more consistency with respect to the chosen feature than the GRNN method. In all volunteers, the WAMP feature showed the best results—in four volunteers at *s*_*lim*_ = 10 mV, which also showed the lowest mean error rates, and in one at *s*_*lim*_ = 20 mV. The GRNN method showed a preference for the MAV feature in the averaged results. Two volunteers performed better when we used the WAMP feature at *s*_*lim*_ = 10 mV. In one volunteer, WL gave the best results, and in another, RMS. Table 4 lists the optimal settings for both methods per feature, averaged over the volunteers (see Eq (16)), with the corresponding error measures. In the PCA method, WAMP at *s*_*lim*_ = 10 mV clearly performed better. The error measures between the different features were much smaller when we used GRNN, showing a maximum difference in *e*_*c*_ of 0.31 mm between MAV and WAMP at *s*_*lim*_ = 20 mV. The methods showed differences in preferred window lengths, with 300 ms in the PCA method and 50 ms in GRNN. However, influence of window length appeared much more profound in GRNN than in PCA, producing maximum *e*_*c*_ differences of merely 0.02 mm in PCA and no less than 0.68 mm in GRNN.

In all volunteers, we found good correlation coefficients (*ρ*). With the PCA method, *ρ* ranged between 0.87 and 0.91 and with the GRNN method, *ρ* ranged between 0.86 and 0.94. The mean *ρ* was 0.89 with both methods (see Table 2). Between features, *ρ* ranged between 0.87 and 0.89.

**Table 4:** The mean optimal results (see Eq (16)) by feature obtained with PCA and GRNN and the corresponding settings.

	RMS		MAV		WL		WAMP *s*_*lim*_ = 10 mV		WAMP *s*_*lim*_ = 20 mV	
	PCA	GRNN	PCA	GRNN	PCA	GRNN	PCA	GRNN	PCA	GRNN
*e*_*c*_(mm)	3.31	2.81	3.33	2.78	3.28	2.88	2.76	2.84	3.27	3.09
*e*_*r*_	0.34	0.29	0.34	0.28	0.33	0.29	0.28	0.29	0.33	0.31
*ρ*	0.87	0.89	0.87	0.89	0.87	0.89	0.89	0.89	0.87	0.88
Configuration	Mono	Bi	Mono	Bi	Mono	Mono	Mono	Mono	Mono	Mono
Window (ms)	50	50	50	50	50	50	300	150	200	50
*D*	7	NA	7	NA	9	NA	9	NA	16	NA
*σ*_*v*_	0.05	NA	0.05	NA	0.05	NA	0.05	NA	0.1	NA
*α*	NA	2	NA	2	NA	2	NA	3	NA	2

The optimal PCA dimension *D* ranged between 6 and 17 in our volunteers and showed an optimum in the averaged results at 9. By evaluating the results for all different dimensions, we found that the first four to five principal components had a large influence on shape prediction. When we used eight principal components, the error values reached a plateau, after which only small changes in the errors occurred. When using nine principal components, we found explained variances in the PCA model of 92 to 96% in our volunteers.

We never found the optimal value for *σ*_*v*_ to be zero, which means that the MMSE analysis performed better. We noted a clear trend towards higher *e*_*c*_ values when increasing *σ*_*v*_. A 0.05 increase of *σ*_*v*_ produced a mean error increase of 0.11 (range: 0.10 to 0.13). This trend occurred in four of five volunteers; volunteer 2 was the only one to show a small error decrease as *σ*_*v*_ was raised to 0.25.

The averaged results showed the lowest error at parameter *α* = 2. Only volunteer 2 clearly deviated with the lowest error at *α = 5*. In this volunteer, the error was 0.50 mm larger at *α = 2*, with the other settings unchanged.

When comparing volunteers by values in *e*_*r*_, we found the PCA method to be more consistent than the GRNN method: the former gave a value range of 0.23 to 0.33 and the latter a range of 0.17 and 0.31. Both methods gave a value of 0.28 for *e*_*r*_ with the averaged data.

## Discussion

Our study has shown that it is, indeed, possible to estimate static 3D lip shapes from volunteer-specific sEMG measurements of facial muscles and the digastric muscle. The tested methods, PCA-based nonlinear regression and a modified GRNN, gave comparable results with an average accuracy of about 2.8 mm in five measured volunteers.

In the PCA approach, MMSE performed significantly better than LSE. The MMSE method uses the additional knowledge that the values in the coefficients **b** are uncorrelated, with zero means and with variances that are known from the PCA model [21]. Therefore, the growth of the coefficients is controlled, and a higher PCA dimension can be achieved, which will lead to a more accurate estimation. The corrected RMS error of 2.76 mm and correlation coefficient of 0.89 are promising results. The modified GRNN method produced almost identical results. Both models seem generally applicable, but PCA was more consistent between volunteers, whereas in four volunteers, the modified GRNN method produced more accurate position estimates.

A disadvantage of using the modified GRNN method could be the fact that GRNN can be regarded as an interpolation method with a lookup-table that is filled by the training set and probed by the sEMG feature vector of the unknown pose. The method performs well as long as the feature vector probes in the vicinity of feature vectors in the table, as was the case in this study. The results are less predictable if the feature vector probes in a white area of the lookup-table, which can occur when a pose is adopted that is not present in the training set. As PCA behaves smoother in the untrained regions, this method may be of better use for our ultimate goal: to predict post-treatment function loss, we will need maximum accuracy in predicting not only 3D lip shapes, but also functional movements that result from multiple muscle activations, even though we could never create a volunteer-specific training set that includes all possible poses.

Since most models in literature do not give quantitative values, we have difficulty comparing our results with previous findings. Lucero & Munhall’s finite-element model of the face and lips uses intramuscular facial EMG measurements (ZYG, LLS, DAO, MEN, OOS, OOI, and the depressor labii inferioris and levator anguli oris muscles) [16]. They placed five markers on the lips and estimated vertical displacements and protrusions of these markers, finding mean correlation coefficients of 0.71 and 0.28 for vertical displacement and protrusion, respectively.

Although our methods performed much better than this finite-element approach, our models could only describe phenomena, whereas a FEM could establish a one-to-one correspondence between anatomy and physiology on the one hand and mathematical structures on the other, which renders it more suitable for a practical application to predict post-treatment function loss. Moreover, the FEM approach can include the (nonlinear) dynamics of anatomy and physiology. Some poses, e.g. the pose adopted when articulating the vowel sounds /a/ and /e/, require little persistent muscle activation. When we disregard the muscle activation patterns (and associated sEMG patterns) that produce these poses, it is much harder to distinguish between them.

We cannot draw any decisive conclusions as to an optimal configuration, feature, or window length for processing sEMG signals. The PCA method showed a preference for unipolar sEMG measurements in combination with the WAMP feature at *s*_*lim*_ = 10 mV and calculated over longer time windows (on average, 300 ms). The GRNN method performed best in a bipolar configuration with the MAV feature determined over a 50-ms time window. However, the optimal settings varied between volunteers, especially in the GRNN method. In the PCA method, we did not find a single best setting for window length either, but the effects on the error with PCA were marginal–probably because we evaluated static poses only with an sEMG-feature sequence averaged over three seconds. The GRNN method performed significantly better with smaller window lengths than with larger ones, possibly because small windows have more fluctuations in the sEMG features, thus expanding the covariance matrix and leading to a better kernel coverage in the 8-dimensional feature space. For the purpose of this study, a volunteer-specific model coupling sEMG to positions, we did not find it necessary to determine one single best configuration for all volunteers, since the parameters could be fine-tuned during the training process with each volunteer’s individual data. Nevertheless, narrowing down the parameter ranges and evaluating only the best performing features would definitely reduce computation time.

Meltzner et al. used a modified Mel-frequency cepstral coefficients (MFCC) algorithm for feature extraction from sEMG [12,13]. MFCCs are frequently used in automatic speech recognition with acoustical signals. Despite the fact that sEMG signals possess different properties than acoustical signals, Meltzner et al. found that an MFCC algorithm tailored to their needs outperformed the other processing algorithms they tested [12,13]. More recently, Längkvist et al. reviewed the applications of deep learning for feature extraction from time series [23], and Wand & Schultz showed the use of deep neural networks in EMG-based speech recognition [24]. These are interesting topics that might improve our results, but we would probably need much more training data. Since the current accuracy has the same order of magnitude as the observation error, these improvements will be marginal. Future experiments may benefit from the inclusion of advanced feature extraction algorithms like the ones mentioned above in combination with high-density sEMG grids. Staudenmann et al. showed that these grids improved sEMG-based muscle force estimation by some 30% [25]. Another good thing about these grids is that they eliminate the need for precise microelectrode placement.

When tested on our five volunteers, our methods produced satisfying initial results and our models showed comparable accuracy in all volunteers. Despite our relatively small sample size, our results indicate that sEMG of the perioral muscles conveys sufficient information to estimate 3D lip positions, and we have identified important parameters. A larger sample size might reveal that window length, configuration, and feature type also have significant influence on the RMS errors. On the other hand, a large sample size may produce significant differences of small RMS errors, which do not have any practical meaning.

Despite similar performances, we favour the PCA-based regression model because of the advantages discussed above, the possible disadvantages of modified GRNN, and the computational load of the estimators, which is in favour of the PCA method.

It must be noted that training sets are volunteer-specific and cannot be used for the estimation of lip poses of other volunteers. This problem also occurs in EMG-speech recognition, as described by Schultz & Wand [10]. They showed that generic independent-speaker models might be feasible but at the cost of higher error. Meltzner et al. argue that speaker-dependent systems do have practical applications and that the minimal amount of training data necessary per individual is not too big of a burden [12]. For our ultimate goal, these volunteer-specific models are key, as each patient is unique.

Variance in facial muscle anatomy, small muscles and electrodes hampers the exact identification of muscles and electrode locations, which may cause small differences between volunteers in muscle activation measurements or amount of crosstalk picked up in the signals. Lapatki et al. showed there is a high risk of crosstalk in the facial musculature due to cocontraction of adjacent muscles [26]. Even when using high-density grids, crosstalk remains visible, even if it is reduced. We saw coactivation in all volunteers and all poses in varying degrees. Apparently, either the volunteers were not always able to perform isolated muscle contractions, or crosstalk occurred.

Our most important conclusion is that features extracted from facial sEMG can estimate lip shapes in 3D with high accuracy. This finding is an essential step forward in constructing a virtual-therapy model to predict post-treatment function loss. We found our sEMG processing parameters to be generally applicable and could use them in our future application for oral and lip cancer patients, so we will not have to optimise our sEMG parameters for each patient individually.

These settings might not only benefit researchers in the field of silent-speech interfaces, but might also be interesting for researchers in the field of human machine interfaces (HMI)—for instance, in projects like Hamedi et al.’s, who used facial sEMG to classify emotions [7,27]. The results of our study seem promising for controlling machines via HMI with support of up to 13 control commands (the thirteen poses). Our models would be able to classify emotions and present them visually in 3D, too. Moreover, as suggested by Honda et al., the models could produce visual output for physiological vocal-tract models in speech production and speech synthesis. Or they could provide visual feedback in EMG-based speech recognition.

For the development of a virtual-therapy model that could predict functional outcome, the current models should be extended to incorporate dynamics as well as unilateral lip movements. Such extension would require video capturing of the lips and bilateral sEMG measurements.

Our recommendation for future research would be to combine FEM with nonlinear regression and apply the estimation techniques to model the neural activation of simulated muscles instead of lip positions, thus separating activation modelling from dynamic modelling. The first step in developing such an activation model of the lips has been taken.

## Conclusion

This study shows that static 3D lip shapes can be estimated from volunteer-specific sEMG measurements of facial muscles and the digastric muscle. The tested methods—PCA-based nonlinear regression and a modified GRNN—gave comparable results with an average accuracy of about 2.8 mm in the five measured volunteers.
